# Antibiotic Resistance Profiles of Bacterial Strains Isolated from the Roots, Cladodes and Rhizosphere of *Opuntia dillenii* (KerGawl.) Haw (Cactaceae) in the Coastal Zone of Benin

**DOI:** 10.3390/microorganisms14071509

**Published:** 2026-07-10

**Authors:** Yves Kévin Brun, Agossou Damien Pacôme Noumavo, Julien Colombet, Bawa Boya, Bartholomew Saanu Adeleke, Haziz Sina, François Lefort

**Affiliations:** 1 Plants and Pathogens Group, Research Institute Land Nature Landscape, HEPIA Geneva School of Engineering, Architecture and Landscape, HES-SO University of Applied Sciences and Arts Western Switzerland, 150 Route de Presinge, 1254 Jussy, Switzerland; kevin.brun@etu.hesge.ch (Y.K.B.); julien.colombet@hesge.ch (J.C.); 2 Laboratory of Microbiology, Food Technology and Phytopathology, Faculty of Science and Technology, University of Abomey-Calavi, Abomey-Calavi 05 P.O. Box 1604, Benin; 3 Laboratory of Biology and Molecular Typing in Microbiology, Faculty of Science and Technology, University of Abomey-Calavi, Cotonou 04 P.O. Box 1107, Benin; boyabawa@gmail.com (B.B.); sina.haziz@gmail.com (H.S.); 4 Department of Microbiology, Faculty of Natural and Applied Sciences, Federal University of Technology and Environmental Sciences, P.M.B. 001, Iyin-Ekiti 360101, Nigeria; bartholomew.adeleke@futes.edu.ng; 5 Food Security and Safety Niche Area, Faculty of Natural and Agricultural Sciences, North-West University, Private Bag X2046, Mmabatho 2735, South Africa

**Keywords:** *Opuntia dillenii*, plant microbiota, multidrug resistance, antibiotic resistance, Benin

## Abstract

Endophytic and rhizosphere bacteria have considerable potential as bioinoculants for enhancing plant growth and improving soil health. However, despite their agricultural importance, their antibiotic resistance profiles remain poorly documented. This study assessed the antibiotic resistance of 31 bacterial strains isolated from the roots, cladodes, and rhizosphere of *O. dillenii* growing in the coastal zone of Benin. Antibiotic susceptibility was evaluated using the Kirby–Bauer disk diffusion method according to CA-SFM/EUCAST guidelines. Depending on the bacterial genus, each strain was tested against 7 to 12 antibiotics selected from a panel of 19 antibiotics representing 14 antimicrobial classes. The results showed moderate resistance of 40–60% for linezolid and clindamycin. Low resistance of 10–39% was recorded for erythromycin and vancomycin. In contrast, the bacterial resistance to Tetracycline, Amoxicillin, Ciprofloxacin, Fusidic Acid, Gentamicin, Quinupristin–dalfopristin, Rifampicin, and Trimethoprim–Sulfamethoxazole remained extremely low (<10%). No bacterial resistance was detected against Ampicillin, Amoxicillin–clavulanic Acid, Ceftriaxone, Cefoxitin, Cefotaxime, Norfloxacin, or Imipenem. Overall, 9 out of 31 endophytic strains (29.03%) exhibited multidrug resistance. Therefore, this finding provides valuable biosafety data for the selection and registration of bacterial biofertilizers and identifies several promising strains for future agricultural applications.

## 1. Introduction

In recent years, interest in the use of microorganism-based products as bioinoculants and/or biological agents has continued to grow. However, their application faces numerous challenges, ranging from laboratory trials to field conditions [1]. Plant growth-promoting bacteria (PGPB) constitute a polyphyletic group of bacteria that interact closely or more loosely with plant roots within the rhizosphere biotope [2]. The most commonly reported PGPB genera include *Bacillus*, *Azoarcus*, *Pseudomonas*, *Serratia*, *Enterobacter*, *Streptomyces*, *Herbaspirillum*, *Azospirillum*, *Azotobacter*, *Klebsiella*, *Alcaligenes*, *Arthrobacter*, *Burkholderia*, *Microbacterium*, *Micrococcus*, *Pantoea*, and *Stenotrophomonas* [3]. Recent scientific advancements in the use of PGPB as bioinoculants have been documented to enhance plant growth and stress tolerance by improving nutrient uptake, photosynthetic efficiency, antioxidant defense stimulation, and phytohormone modulation through direct and indirect mechanisms [4].

Despite their agronomic benefits, PGPB raise legitimate concerns regarding antibiotic resistance. Some strains may harbor antibiotic resistance genes (ARGs), which can be transmitted vertically within the same species or horizontally between different bacterial species via mobile genetic elements (MGEs), such as plasmids, transposons, and integrons [5]. The spread of ARGs through horizontal transfer can contribute to the emergence of multidrug-resistant bacteria, commonly known as ‘superbugs’, reducing the effectiveness of antibiotics and posing a serious threat to human and animal health [5,6]. In agricultural systems, including coastal agroecosystems, soils and plants can act as reservoirs of ARGs, particularly when microbial inoculants are applied without thorough biosafety assessment. ARGs present in microorganisms in the soil can be transferred to bacteria associated with plant roots and above-ground parts, including edible tissues, thereby facilitating their potential entry into the food chain and the human microbiota [7,8]. Consequently, the evaluation of biofertilizers must include ARG resistance and transfer analyses, as well as the exclusion of multidrug-resistant or pathogenic strains [2].

The use of plant-associated bacteria as biofertilizers, therefore, requires two types of assessment: their agronomic performance and a detailed characterization of their antibiotic resistance profiles [9]. Several studies have shown that microorganisms associated with plants in an agricultural context may harbor antibiotic-resistant or multidrug-resistant bacteria [10,11,12]. Despite the research findings on the bacteria inhabiting conventional crops, the research data on the antibiotic resistance profiles of bacteria associated with wild plants adapted to harsh environments are limited. Consequently, the biosafety status of the bacterial communities occupying these ecological niches remains insufficiently documented, constituting a significant gap in current knowledge.

Plants naturally adapted to extreme environments often harbor specialized microbial communities that contribute to host survival under adverse conditions. These microorganisms may possess genetic, physiological, and metabolic adaptations that enable them to thrive under conditions such as high salinity, drought, extreme temperatures, nutrient limitation, and pathogen pressure. Owing to these unique adaptive traits, plant-associated microbes from extreme habitats are increasingly recognized as valuable reservoirs of novel PGPB with considerable potential for sustainable agricultural applications [13].

*Opuntia dillenii* (Ker Gawl.) Haw. is a perennial cactus widely distributed along the coast of Benin. This species is naturally adapted to harsh environmental conditions, including high salinity, water stress, and poor nutrient availability. Consequently, it may harbor specialized microbial communities exhibiting unique functional characteristics and potential relevance to sustainable agriculture [14].

In a previous study, Brun et al. [3] isolated and characterized 31 endophytic and rhizosphere bacterial strains associated with *O. dillenii* in the coastal region of Benin. These strains, belonging to diverse bacterial genera, exhibited several plant growth-promoting traits, including phosphate solubilization, siderophore production, nitrogen fixation, and indole-3-acetic acid production, highlighting their potential use as biofertilizers. In addition, several isolates demonstrated antagonistic activity against *Sclerotium rolfsii*, a major soil-borne plant pathogen, both under in vitro and greenhouse conditions [15]. Some strains also promoted plant growth in the absence of pathogen pressure.

Collectively, the finding suggests that bacteria associated with *O. dillenii* represent promising candidates for sustainable agricultural applications. However, despite their demonstrated plant growth-promoting and biocontrol potential, information regarding their biosafety remains lacking. In particular, their antibiotic resistance profiles have not yet been characterized, which is fundamental for the safe development and registration of microbial inoculants. Therefore, before any agricultural application can be considered, the safety of these bacterial strains must be thoroughly assessed.

Hence, the present study aimed to characterize the antibiotic resistance profiles of endophytic and rhizosphere bacterial strains associated with *O. dillenii* and identify multidrug-resistant isolates. Beyond addressing a critical biosafety requirement, this work expands current knowledge on the resistome of plant-associated bacteria inhabiting extreme coastal environments and provides a scientific basis for the development of safe microbial biofertilizers adapted to stress-prone agroecosystems.

## 2. Materials and Methods

### 2.1. Bacterial Strains

The bacterial strains used in this study were isolated from the endophytic tissues and rhizosphere of *O. dillenii*, collected in the coastal zone of Benin. The bacterial isolates used in this study were previously characterized and stored at −20 °C in nutrient broth (HIMEDIA, Mumbai, India) supplemented with 30% glycerol [3]. A total of 31 bacterial strains were used for all analyses (Table 1).

### 2.2. Antibiotic Susceptibility Test

**Figure 1:**
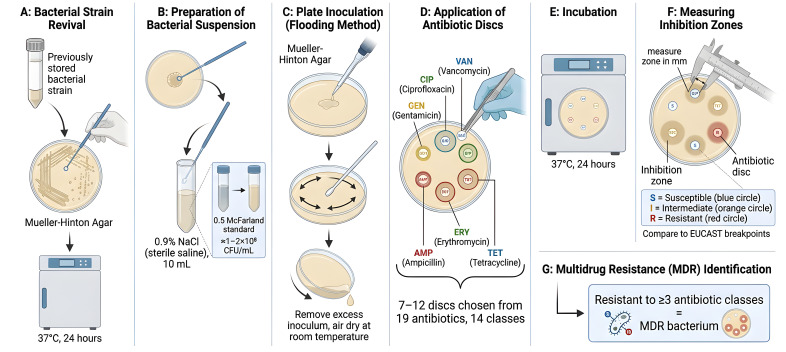
Workflow of antibiotic susceptibility test and multidrug resistance (MDR) determination of bacterial strains isolated from *O. dillenii*.

Antibiotic susceptibility test was performed using the Kirby–Bauer disk diffusion method according to EUCAST guidelines [16], as shown in Figure 1. The previously stored strains were revived by inoculation onto Mueller–Hinton Agar (HIMEDIA, India) and then incubated at 37 °C for 24 h in the incubator. To do this, each strain was suspended in 10 mL of sterile 0.9% saline solution (NaCl, *w*/*v*), maintaining a turbidity of 0.5 McFarland (≈1–2 × 10^8^ CFU/mL). The plates were inoculated using the flooding method. Excess inoculum was removed before drying at room temperature. The antibiotic discs were aseptically applied onto the surface of the inoculated agar, and the plates were incubated at 37 °C for 24 h.

Between 7 and 12 antibiotics, out of a choice of nineteen antibiotics belonging to fourteen classes were tested: Glycopeptides (Vancomycin, 30 µg), Quinolones (Norfloxacin, 10 µg, and Ciprofloxacin, 5 µg), lincosamides (clindamycin, 2 µg), tetracyclines (Tetracycline, 30 µg), Cephalosporins (Cefoxitin, 30 µg; Cefotaxime, 30 µg, and Ceftriaxone, 30 µg), Oxazolidinones (linezolid, 30 µg), aminoglycosides (Gentamicin, 10 µg), Penicillins and β-lactamase inhibitors (Ampicillin, 10 µg; Amoxicillin, 25 µg, and Amoxicillin–clavulanic acid, 30 µg), Streptogramins (Quinupristin–dalfopristin, 15 µg), Carbapenems (Imipenem, 10 µg), macrolides (Erythromycin, 15 µg), sulfonamides (Trimethoprim–sulfamethoxazole, 25 µg), fusidic acid (Fusidic acid, 10 µg), and Rifamycins (Rifampicin, 5 µg). The diameters of the inhibition zones were measured and compared to the reference values in the Clinical Breakpoint Table to classify the strains as susceptible (S), intermediate (I), or resistant (R) [17]. For bacteria without specific breakpoints, comparisons were made using clinical breakpoints for phylogenetically related organisms (Table 2). Subsequently, multidrug-resistant (MDR) bacteria were identified. Multidrug resistance was defined as resistance to at least three classes of antibiotics, in accordance with the classification criteria described by Magiorakos et al. [18]. Only resistance (R) was considered. Strains with intermediate (I) profiles were not considered in this classification. Antibiotic susceptibility testing was conducted in triplicate for each bacterial strain.

### 2.3. Statistical Analysis

Statistical analysis was performed using R (version 4.4.3). The ggplot2 package (version 4.0.1) was used to create the Heatmaps.

## 3. Results

### 3.1. Antibiotic Susceptibility Profile of Bacteria

**Figure 2:**
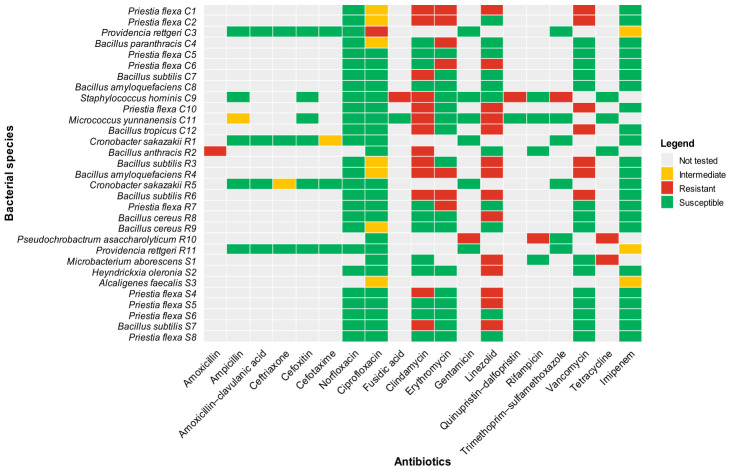
Heatmap showing the antibiotic susceptibility profile of the bacteria.

All strains are susceptible or intermediate to at least one of the antibiotics tested (Figure 2). Therefore, no strain is completely resistant to all the antibiotics tested. Overall, moderate resistance levels (40–60%) were observed for linezolid and clindamycin. Low resistance levels (10–39%) were recorded for erythromycin and vancomycin. In contrast, resistance to tetracycline, amoxicillin, Ciprofloxacin, Fusidic acid, Gentamicin, Quinupristin–dalfopristin, Rifampicin, and Trimethoprim–sulfamethoxazole remained extremely low (<10%). No resistance was detected against Ampicillin, Amoxicillin–clavulanic acid, Ceftriaxone, Cefoxitin, Cefotaxime, Norfloxacin, or Imipenem (Table 3).

### 3.2. Genus-Specific Patterns of Antibiotic Resistance

**Figure 3:**
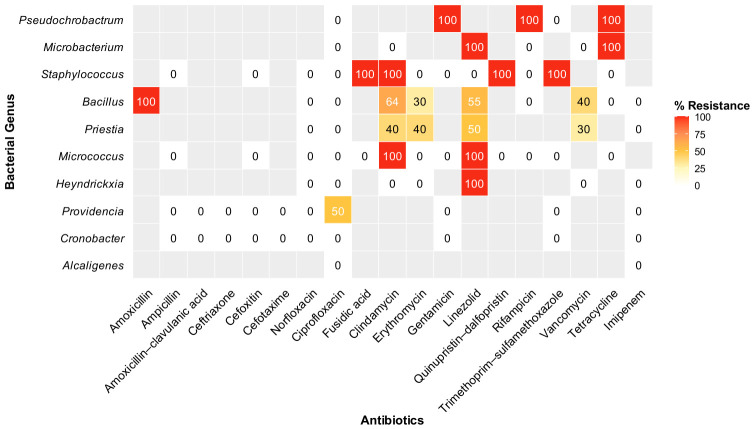
Heatmap showing the distribution of antibiotic resistance across bacterial genera. Resistance values are expressed as the percentage of isolates resistant to each antibiotic. Genera tested for similar antibiotics are grouped. Gray cells indicate combinations for which no testing was performed.

A detailed analysis of resistance rates by bacterial genus and antibiotic reveals marked heterogeneity within the bacterial community studied (Figure 3). The genus *Bacillus* exhibits the highest levels of resistance to several antibiotics. High resistance is observed for Amoxicillin (100%) and Clindamycin (63.6%), and moderate resistance for linezolid (54.5%) and Vancomycin (40%). In contrast, no resistance was observed for Norfloxacin, Ciprofloxacin, Rifampicin, Tetracycline, and Imipenem. The genus *Priestia* shows moderate resistance to Clindamycin (40%), Erythromycin (40%), and Linezolid (50%), and low resistance to vancomycin (30%). However, all *Priestia* strains are susceptible to Norfloxacin, Ciprofloxacin, and Imipenem. Conversely, the *Cronobacter* and *Alcaligenes* genera are distinguished by a complete absence of resistance to all tested antibiotics. The *Providencia* genus remains predominantly susceptible to the tested antibiotics, except for Ciprofloxacin (50%). The minority genera, meanwhile, exhibit more specific resistance profiles: *Staphylococcus* shows 100% resistance to Fusidic acid, Clindamycin, Quinupristin–dalfopristin, and Trimethoprim–sulfamethoxazole; *Micrococcus* is resistant to Clindamycin and Linezolid (100%); *Pseudochrobactrum* exhibits high resistance to Gentamicin, Rifampicin, and Tetracycline (100%); while *Microbacterium* is resistant to Linezolid and Tetracycline (100%), and *Heyndrickxia* is resistant only to Linezolid (100%).

### 3.3. Distribution and Prevalence of Multidrug-Resistant Bacterial Strains

**Figure 4:**
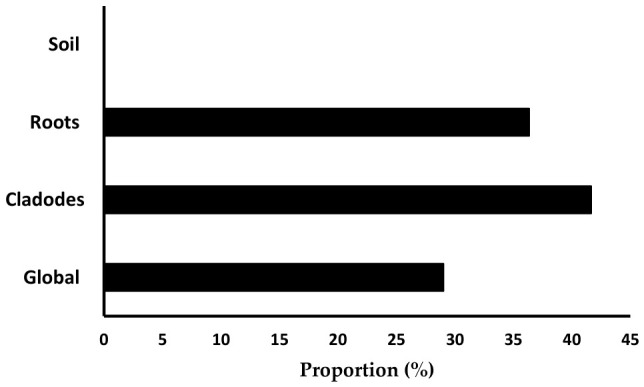
Proportion of multidrug-resistant bacteria isolated from *O. dillenii*.

Among the 31 strains tested, 9 (29.03%) were identified as multidrug-resistant (MDR). These MDR strains were all endophytic and included *P. flexa* C1, *P. flexa* C2, *P. flexa* C10, *S. hominis* C9, *B. tropicus* C12, *B. subtilis* R3, *B. subtilis* R6, *B. amyloliquefaciens* R4, and *P. asaccharolyticum* R10. No rhizosphere bacteria were classified as MDR (Figure 4). Conversely, eight isolates, namely *P. flexa* C5, *B. amyloliquefaciens* C8, *C. sakazakii* R1, *C. sakazakii* R5, *P. rettgeri* R11, *A. faecalis* S3, *P. flexa* S6, and *P. flexa* S8, showed either no resistance or only intermediate susceptibility profiles to the antibiotics tested.

## 4. Discussion

Plant growth-promoting bacteria (PGPB) have emerged as promising tools for sustainable agriculture. However, their application as biofertilizers requires a thorough safety assessment, particularly regarding antibiotic resistance [26]. In this study, the antibiotic susceptibility profiles of 31 endophytic and rhizosphere bacterial strains associated with *O. dillenii* were characterized. The majority of the antibiotics tested showed very low or no resistance, indicating a low overall level of antibiotic resistance among the bacteria isolated from *O. dillenii*. The absence of resistance to Imipenem, as well as to the third-generation Cephalosporins tested (Ceftriaxone and Cefotaxime), is particularly noteworthy, as these compounds belong to antimicrobial classes designated by the World Health Organization as Highest Priority Critically Important Antimicrobials (HPCIA) for human medicine [27].

The resistance patterns observed in *Bacillus* and *Priestia* in the present study were consistent with those reported by Dhanokar et al. [28] in endophytic bacteria isolated from *Mesosphaerum suaveolens*, *Azadirachta indica*, *Zingiber officinale*, *Allium sativum*, and *Aloe barbadensis*. These authors identified *B. licheniformis*, *B. stercoris*, *B. tequilensis*, *B. subtilis*, *B. rugosus*, and *B. pumilus* together with *Priestia megaterium* and *P. flexa*. The highest levels of resistance were observed among *Bacillus* isolates, with frequencies reaching 56.25% for Amoxicillin–clavulanic acid, 50% for Azithromycin, and 43.75% for Ceftazidime. However, whereas Dhanokar et al. [28] reported resistance primarily to β-lactams and macrolides, the profiles observed in the present study also included resistance to lincosamides, oxazolidinones, and glycopeptides. In contrast, no resistance to Imipenem or Fluoroquinolones was detected among isolates belonging to either genus. The recurrent detection of *Bacillus* and *Priestia* among antibiotic-resistant endophytic bacteria associated with taxonomically distant plant species suggests that these genera constitute important members of plant-associated bacterial communities. Nevertheless, the differences observed between the two studies indicate that resistance profiles are strongly influenced by the host plant species, environmental conditions, and the specific bacterial strains involved.

Tran et al. [29] showed that root exudates can promote the accumulation of antibiotic resistance genes and mobile genetic elements in the rhizosphere. This observation is particularly relevant to the present study, as the two *B. amyloliquefaciens* isolates displayed contrasting susceptibility profiles. Isolate R4, recovered from roots, exhibited a multidrug-resistant phenotype, whereas isolate C8, isolated from cladodes, was susceptible to all antibiotics tested. Although this difference may reflect the influence of the colonized plant compartment, the available data do not allow a causal relationship between rhizosphere conditions and the observed resistance phenotype to be established. Similarly, Joly et al. [30] reported the absence of antibiotic resistance genes in strain B25 of the *B. velezensis/B. Methylotrophicus* group, a characteristic supporting its use in agricultural applications. Together, these observations highlight the importance of strain-level evaluation, as substantial differences may occur among isolates belonging to the same bacterial species.

In contrast to the Bacillaceae isolates, strains belonging to the genera *Cronobacter* and *Alcaligenes* showed no resistance to the antibiotics tested, although certain species within these genera are recognized as opportunistic human pathogens [31,32]. The *Providencia* isolate was likewise susceptible to most antibiotics tested, with resistance observed only to ciprofloxacin. In contrast, *Staphylococcus* exhibited the most pronounced resistance profiles among the less frequently represented genera. Given that several *Staphylococcus* species are important human pathogens, the occurrence of such resistance phenotypes warrants particular attention. These resistance traits may reflect previous exposure to environmental or anthropogenic selective pressures; however, their precise origin cannot be determined from the data generated in this study alone [33,34]. Beyond their antibiotic susceptibility profiles, the detection of these genera within the *O. dillenii* microbiome also deserves consideration from a One Health perspective. Cechin et al. [35] emphasized that plant-associated bacteria may constitute a potential route of human exposure through the consumption of fresh plant products.

The multidrug resistance profiles observed are limited exclusively to endophytic bacteria from phylogenetically diverse genera, whereas no strains from the rhizosphere exhibit such a phenotype. This profile reveals a significant spatial structuring of resistance. Indeed, the literature shows that endophytes, due to their close contact with plant tissues, are more likely to accumulate multiple resistances compared to rhizobacteria, as demonstrated by Sarkar et al. [11], who described up to 53% MDR strains among endophytic bacteria of medicinal plants. Similarly, Patra et al. [36] observed that an endophytic *Exiguobacterium* strain isolated from carrots grown in amended soil contained several ARGs, coupled with resistance to beta-lactams, fluoroquinolones, and aminoglycosides. Ultimately, the restriction of multidrug resistance phenotypes to endophytic isolates suggests a possible compartment-specific structuring of resistance within internal plant tissues. This spatial pattern suggests that microbiological risk assessments based solely on rhizosphere analysis may lead to sampling bias, potentially overlooking resistant bacterial populations occurring within internal plant tissues.

One limitation of this study is the exclusive use of a phenotypic approach to antibiotic resistance. In the absence of molecular analyses, it was not possible to distinguish between intrinsic and acquired resistance or to identify the underlying genetic determinants. Furthermore, the mobility and transfer potential of the resistance traits observed could not be assessed. Consequently, the resistance profiles reported here should be interpreted with caution, as phenotypic resistance does not necessarily reflect the underlying molecular mechanisms. Further studies incorporating the detection of resistance and virulence genes, as well as the characterization of mobile genetic elements, are needed to better assess the safety of the strains investigated.

Beyond these methodological limitations, the occurrence of resistant and multidrug-resistant isolates deserves particular attention from a biosafety perspective. Although resistance was detected in only a limited number of strains, the use of plant growth-promoting bacteria as biofertilizers implies their deliberate introduction into agricultural environments. Such introductions may contribute to the persistence and dissemination of antimicrobial resistance determinants within plant-associated and soil microbial communities. If transferable resistance genes are involved, their spread to other environmental or clinically relevant bacteria could contribute to the broader dissemination of antimicrobial resistance. Given that antimicrobial resistance is recognized as a major global public health challenge and may compromise the effectiveness of antibiotic therapies, the evaluation of resistance profiles should be considered an essential criterion in the selection of bacterial strains intended for agricultural applications.

## 5. Conclusions

This study revealed a generally low level of antibiotic resistance among bacteria associated with *O. dillenii*, although marked differences were observed between bacterial genera and even between strains of the same species. Resistance phenotypes were concentrated mainly within Bacillaceae, whereas several opportunistic genera remained fully susceptible to the antibiotics tested. Multidrug-resistant profiles were detected exclusively among endophytic isolates, highlighting the importance of considering internal plant tissues when assessing plant-associated resistomes. These findings emphasize the need for strain-level biosafety evaluation before the agricultural application of plant growth-promoting bacteria and support the relevance of a One Health perspective in the monitoring of antibiotic resistance within plant microbiomes.

## Figures and Tables

**Table 1:** Origin, taxonomic identification, plant growth-promoting traits, and Gram reaction of bacterial strains isolated from cladodes, roots, and rhizosphere of *O. dillenii*.

Origin/Source	Codes	GenBank Accession No.	Identity	Functional Characteristics	Gram
Cladode	C1	PZ149653	*Priestia flexa*	Nitrogen fixation, phosphate solubilization, protease, and amylase activity	G+
	C2	PZ149654	*Priestia flexa*	Nitrogen fixation, phosphate solubilization, protease, and amylase activity	G+
	C3	PZ102197	*Providencia rettgeri*	Siderophore, IAA production, and amylase activity	G−
	C4	PZ149655	*Bacillus paranthracis*	Siderophore production, phosphate solubilization, IAA production, and protease activity	G+
	C5	PZ149656	*Priestia flexa*	EPS production, nitrogen fixation, phosphate solubilization, IAA production, protease, and lipase activity	G+
	C6	PZ149657	*Priestia flexa*	EPS production, nitrogen fixation, IAA production, protease, and lipase activity	G+
	C7	PZ149658	*Bacillus subtilis*	EPS production, nitrogen fixation, siderophore production, phosphate solubilization, IAA production, and protease activity	G+
	C8	PZ149659	*Bacillus amyloliquefaciens*	EPS production, nitrogen fixation, siderophore production, phosphate solubilization, IAA production, protease, and lipase activity	G+
	C9	PZ102203	*Staphylococcus hominis*	Nitrogen fixation, siderophore production, phosphate solubilization, and IAA production	G+
	C10	PZ149660	*Priestia flexa*	Nitrogen fixation, siderophore production, phosphate solubilization, IAA production, and protease activity	G+
	C11	PZ102205	*Micrococcus yunnanensis*	EPS production, nitrogen fixation, siderophore production, phosphate solubilization, IAA production, and amylase activity	G+
	C12	PZ149661	*Bacillus tropicus*	Phosphate solubilization, IAA production, and protease activity	G+
Root	R1	PZ102207	*Cronobacter sakazakii*	EPS production, nitrogen fixation, and siderophore production	G−
	R2	PZ149662	*Bacillus anthracis*	Siderophore production, phosphate solubilization, protease, and lipase activity	G+
	R3	PZ149663	*Bacillus subtilis*	EPS production, nitrogen fixation, siderophore production, phosphate solubilization, protease, and lipase activity	G+
	R4	PZ149664	*Bacillus amyloliquefaciens*	EPS production, nitrogen fixation, siderophore production, phosphate solubilization, protease, and lipase activity	G+
	R5	PZ102211	*Cronobacter sakazakii*	EPS production, nitrogen fixation, and siderophore production	G−
	R6	PZ149665	*Bacillus subtilis*	EPS production, nitrogen fixation, siderophore production, phosphate solubilization, protease, lipase, and amylase activity	G+
	R7	PZ149666	*Priestia flexa*	Nitrogen fixation, siderophore production, phosphate solubilization, protease, and amylase activity	G+
	R8	PZ149667	*Bacillus cereus*	Protease and amylase activity	G+
	R9	PZ149668	*Bacillus cereus*	Siderophore production, phosphate solubilization, protease, and lipase activity	G+
	R10	PZ102216	*Pseudochrobactrum asaccharolyticum*	Siderophore production and phosphate solubilization	G−
	R11	PZ102217	*Providencia rettgeri*	Siderophore production, IAA production, and amylase activity	G−
Soil	S1	PZ102218	*Microbacterium aborescens*	Siderophore production, phosphate solubilization, IAA production, and protease activity	G+
	S2	PZ102219	*Heyndrickxia oleronia*	Siderophore production, phosphate solubilization, and lipase activity	G+
	S3	PZ102220	*Alcaligenes faecalis*	Phosphate solubilization	G−
	S4	PZ149669	*Priestia flexa*	Nitrogen fixation, phosphate solubilization, and protease activity	G+
	S5	PZ149670	*Priestia flexa*	Nitrogen fixation, phosphate solubilization, protease, and amylase activity	G+
	S6	PZ149671	*Priestia flexa*	Nitrogen fixation, phosphate solubilization, protease, and amylase activity	G+
	S7	PZ149672	*Bacillus subtilis*	EPS production, nitrogen fixation, siderophore production, phosphate solubilization, protease, and lipase activity	G+
	S8	PZ149673	*Priestia flexa*	Nitrogen fixation, siderophore production, phosphate solubilization, protease, and amylase activity	G+

Key: EPS: exopolysaccharide production; IAA: indole-3-acetic acid production; G+: Gram-positive; G−: Gram-negative.

**Table 2:** EUCAST clinical breakpoints are applied to interpret the results of antimicrobial susceptibility testing for different bacterial strains.

Genus	Available Breakpoints		Critical Breakpoints Used	Quality Control Strains	Reason for Selection
	Yes	No			
*Priestia* spp.		✓	*Bacillus* spp.	*Staphylococcus aureus* ATCC 29213	Reclassified from *Bacillus* spp. [19]
*Bacillus* spp.	✓		*Bacillus* spp.	*Staphylococcus aureus* ATCC 29213	-
*Staphylococcus* spp.	✓		*Staphylococcus* spp.	*Staphylococcus aureus* ATCC 29213	-
*Micrococcus* spp.		✓	*Staphylococcus* spp.	*Staphylococcus aureus* ATCC 29213	Closely related to *Staphylococcus* (Family Micrococcaceae) [20,21]
*Cronobacter* spp.	✓		Enterobacterales	*E. coli* ATCC 25922	-
*Providencia* spp.	✓		Enterobacterales	*E. coli* ATCC 25922	-
*Microbacterium* spp.		✓	*Corynebacterium* spp.	*Staphylococcus aureus* ATCC 29213	Taxonomically related to *Corynebacterium* (Phylum *Actinobacteria*) [22,23]
*Heyndrickxia* spp.		✓	*Bacillus* spp.	*Staphylococcus aureus* ATCC 29213	Reclassified from *Bacillus* spp. [19,24]
*Alcaligenes* spp.		✓	*Pseudomonas* spp.	*Pseudomonas aeruginosa* ATCC 27853	Taxonomically related to *Pseudomonas* [20,25]
*Pseudochrobactrum* spp.		✓	Enterobacterales	*E. coli* ATCC 25922	In the absence of EUCAST breakpoints for this genus, Enterobacterales values were used.

**Table 3:** Antibiotic resistance frequency and resistance level among the bacterial isolates.

Class	Antibiotic	Resistant Isolates (n)	Resistance Rate (%)	Resistance Level
Oxazolidinones	Linezolid	14	45.16	Moderate resistance
Lincosamides	Clindamycin	13	41.94	
Macrolides	Erythromycin	7	22.58	Low resistance
Glycopeptides	Vancomycin	7	22.58	
Tetracyclines	Tetracycline	2	6.45	Extremely low resistance
Penicillins and β-lactamase inhibitors	Amoxicillin	1	3.23	
Quinolones	Ciprofloxacin	1	3.23	
Fusidic acid	Fusidic acid	1	3.23	
Aminoglycosides	Gentamicin	1	3.23	
Streptogramins	Quinupristin–dalfopristin	1	3.23	
Rifamycins	Rifampicin	1	3.23	
Sulfonamides	Trimethoprim–sulfamethoxazole	1	3.23	
Penicillins and β-lactamase inhibitors	Ampicillin	0	0.00	No resistance
Penicillins and β-lactamase inhibitors	Amoxicillin–clavulanic acid	0	0.00	
Cephalosporins	Ceftriaxone	0	0.00	
Cephalosporins	Cefoxitin	0	0.00	
Cephalosporins	Cefotaxime	0	0.00	
Quinolones	Norfloxacin	0	0.00	
Carbapenems	Imipenem	0	0.00	

Resistance level: no resistance (0%), extremely low resistance (<10%), low resistance (10–39%), moderate resistance (40–60%), high resistance (61–80%), and very high resistance (>80%).

## Data Availability

The original contributions presented in this study are included in the article. Further inquiries can be directed to the corresponding authors.
